# Cannabidiol‐induced activation of the metallothionein pathway impedes anticancer effects of disulfiram and its metabolite CuET

**DOI:** 10.1002/1878-0261.13114

**Published:** 2021-10-26

**Authors:** Tereza Buchtova, Zdenek Skrott, Katarina Chroma, Jiri Rehulka, Petr Dzubak, Marian Hajduch, David Lukac, Stefanos Arampatzis, Jiri Bartek, Martin Mistrik

**Affiliations:** ^1^ Faculty of Medicine and Dentistry Institute of Molecular and Translational Medicine Palacky University Olomouc Czech Republic; ^2^ Danish Cancer Society Research Center Copenhagen Denmark; ^3^ Division of Genome Biology Department of Medical Biochemistry and Biophysics Science for Life Laboratory Karolinska Institute Stockholm Sweden

**Keywords:** cancer, cannabidiol, CuET, disulfiram, metallothionein

## Abstract

Disulfiram (DSF), an established alcohol‐aversion drug, is a candidate for repurposing in cancer treatment. DSF’s antitumor activity is supported by preclinical studies, case reports, and small clinical trials; however, ongoing clinical trials of advanced‐stage cancer patients encounter variable results. Here, we show that one reason for the inconsistent clinical effects of DSF may reflect interference by other drugs. Using a high‐throughput screening and automated microscopy, we identify cannabidiol, an abundant component of the marijuana plant used by cancer patients to mitigate side effects of chemotherapy, as a likely cause of resistance to DSF. Mechanistically, in cancer cells, cannabidiol triggers the expression of metallothioneins providing protective effects by binding heavy metal‐based substances including the bis‐diethyldithiocarbamate‐copper complex (CuET). CuET is the documented anticancer metabolite of DSF, and we show here that the CuET’s anticancer toxicity is effectively neutralized by metallothioneins. Overall, this work highlights an example of undesirable interference between cancer therapy and the concomitant usage of marijuana products. In contrast, we report that insufficiency of metallothioneins sensitizes cancer cells toward CuET, suggesting a potential predictive biomarker for DSF repurposing in oncology.

## Graphical abstract

A complex of copper and dithiocarbamate, CuET, a product of disulfiram (DSF) metabolization, targets cancer cells via proteotoxic stress and DNA damage. Concomitant usage of cannabis‐derived products attenuates the effect of CuET by promoting the induction of metallothioneins, which then bind to CuET and interfere with its cellular activities.

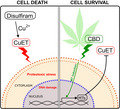

**Abbreviations**
5‐HT1A: 5‐hydroxytryptamine receptor subtype 1A
A2A: adenosine A2A receptor
BCDS: bathocuproine disulfonic acid
CB1: cannabinoid receptor 1
CB2: cannabinoid receptor 2
CBD: cannabidiol
Cd: cadmium
CuET: bis‐diethyldithiocarbamate‐copper complex
DSF: disulfiram
GAPDH: glyceraldehyde 3‐phosphate dehydrogenase
GPR55: G protein‐coupled receptor 55
HPLC‐MS: high‐pressure liquid chromatography‐mass spectrometry
MTF1: metal transcriptional factor 1
MTs: metallothioneins
NPL4: nuclear protein localization protein 4
PPARγ: peroxisome proliferator‐activated receptor γ
PRISM: profiling relative inhibition simultaneously in mixtures
qPCR: quantitative polymerase chain reaction
TRPV1: transient receptor potential cation channel subfamily V members 1
TRPV2: transient receptor potential cation channel subfamily V members 2
Ub: ubiquitin
WB: western blotting
Zn: zinc

## 1 Introduction

Disulfiram (Antabuse), a drug used for almost 70 years to treat alcohol abuse, is an emerging candidate for repurposing in cancer therapy. Antitumor activity of disulfiram (DSF) is supported by numerous preclinical studies, case reports, and small clinical trials [1, 2, 3], yet clinical data from larger randomized trials are still lacking. Despite several promising case reports about durable remissions of advanced‐stage cancer patients after DSF therapy [1, 4, 5], results from clinical trials are still limited and less favorable [6], a trend that is shared with other repurposed drugs [7]. The results from the few clinical trials available so far suggest that DSF´s anticancer effect may be limited to a subset of cancer patients [8, 9], thereby raising a need for the identification of biomarkers that would help guide the patient selection in the future. A broader assessment of DSF in clinical oncology had been hindered mainly by the unknown identity of the active anticancer metabolite and its mechanism of action in cancer cells, including the key molecular target. Consequently, there is currently no reliable way to predict who among cancer patients is likely to benefit from the DSF treatment. In an effort to improve this situation, we have recently discovered that DSF is metabolized in the human body to bis‐diethyldithiocarbamate‐copper complex (CuET), that CuET represents the long‐sought‐after active compound that kills cancer cells, and that mechanistically, such toxicity to cancer cells reflects CuET‐mediated impairment of NPL4, an essential cofactor of p97 segregase broadly involved in the degradation of cellular proteins [10, 11]. We have also noticed that the CuET complex levels assessed after administration of the same dose of DSF vary significantly among patients [10]. We hypothesize that the observed variable clinical responses to DSF treatment might be attributable, at least in part, to the divergent extent of CuET formation, a process that is likely influenced by genetic and environmental factors, the latter including copper intake and the overall diet. Furthermore, the effectiveness of DSF treatment may be affected also by factors such as concomitant exposure to other drugs or pharmaceutically active compounds, a scenario particularly likely for advanced‐stage cancer patients. With the primary mechanism of anticancer activity of DSF known, the identification of such factors that impact cellular responses to DSF/CuET is key to facilitate the repurposing of DSF in clinical oncology.

In this study, we identified cannabidiol (CBD), the most abundant nonpsychoactive compound and the second most abundant cannabinoid from the *Cannabis* sp. plant (known as marijuana), as a compound, strongly interfering with the anticancer activity of CuET. Apart from recreational use, marijuana and its products have been advocated for the treatment of a range of inflammatory, autoimmune and neurodegenerative conditions, epilepsy, multiple sclerosis, arthritis, and schizophrenia [12, 13, 14]. Mechanistically, CBD shows a low affinity for both cannabinoid receptors: CB1 and CB2, and while CBD exerts negative allosteric modulatory effects on CB1, it is an agonist of CB2. In addition to cannabinoid receptors, other potential targets of CBD have been reported, such as the transient receptor potential cation channel subfamily V members 1 and 2 (TRPV1/2; agonist), peroxisome proliferator‐activated receptor γ (PPARγ; agonist), G protein‐coupled receptor 55 (GPR55; antagonist), 5‐hydroxytryptamine receptor subtype 1A (5‐HT1A, agonist), and adenosine A2A receptor (A2A, agonist) [12, 13, 14, 15].

Notably, CBD is popular among cancer patients due to its ability to reduce the adverse effects of chemotherapy, including vomiting, nausea, and weight loss [16, 17]. Various anticancer effects were also reported for cannabinoids including antiproliferative and proapoptotic properties, interference with angiogenesis, cancer cell migration, adhesion, and invasion [12, 18, 19], a notion which further motivates cancer patients to use CBD.

## 2 Materials and methods

### 2.1 Cell lines

Human osteosarcoma U‐2‐OS (ATCC), human breast adenocarcinoma MDA‐MB‐231 (ATCC), U‐2‐OS ectopically expressing NPL4‐GFP [10], U‐2‐OS ectopically expressing MT‐2A‐GFP, and retinal pigment epithelia hTERT RPE‐1 (ATCC) were maintained in DMEM (Lonza, Basel, Switzerland) supplemented with 10% fetal bovine serum (Thermo Fisher Scientific, Waltham, MA, USA) and 1% penicillin/streptomycin (Sigma‐Aldrich, St. Louis, MO, USA). MCF 10A nontransformed human breast epithelial cell line and well‐characterized breast cancer cell lines obtained from the ATCC repository, HCC1954 and SK‐BR‐3 (both HER‐+ve), ZR‐75‐1 and MCF7 (both luminal, ER‐+ve), MDA‐MB‐436, MDA‐MB‐231, CAL‐51, MDA‐MB‐468, HCC70, MDA‐MB‐453, MDA‐MB‐157 (all triple‐negative), used for metallothionein expression analysis were grown all grown under identical conditions in a mixture of 50% MEGM (Lonza) and 50% DMEM (Gibco, Amarillo, TX, USA) media, supplemented with 10% FBS (Gibco), 1% penicillin/streptomycin (Gibco), and SingleQuots supplement kit (Lonza) as recommended by the manufacturer.

### 2.2 High‐throughput screening for CuET activity interferers

U‐2‐OS‐NPL4‐GFP was seeded into 384‐well plates (PerkinElmer, CellCarrier‐UltraPlate) at a concentration of 1250 cells per well in 30 μL of media. The next day, the cells were pretreated with selected 1282 compounds (see enclosed Table S1) overnight (17 h). The used concentration for each of the compounds was set to 10 μm. Subsequently, the cells were treated with 0.2 µm CuET for 3 h to induce aggregation and immobilization of the NPL4‐GFP signal. Next, the cells were pre‐extracted by 0.2% Triton X‐100 buffer with 10 μm Hoechst 33342, washed by PBS, and fixed by 1% formalin for 10 min. After the fixation, the wells were washed by PBS and as the last step, the 30 μL of PBS was dispensed per well. Each well was acquired using an automated microscopic platform (Yokogawa CV7000, 10× air objective), with 4 microscopic fields per well. Images were analyzed by the Columbus image analysis pipeline (PerkinElmer, Waltham, MA, USA). Individual nuclei were recognized based on the Hoechst dye signal. In each nucleus, the level of the NPL4‐GFP signal was scored and means of fluorescence intensity per nucleus were plotted.

### 2.3 RNA interference and overexpression experiments

siRNA against MTF1 (cat. no: SR302991, OriGene) and against MT‐2A (cat. no: SR302987, OriGene) were used. For overexpression plasmids, Myc‐DDK‐tagged MT‐2A (cat. no: RC202748, OriGene) and GFP‐MT‐2A (cat. no: RG202748, OriGene) were used. Transfection of siRNA was performed with Lipofectamine RNAiMAX (cat. n.: 13778‐075, Invitrogen, Waltham, MA, USA) according to the manufacturer's instructions. The plasmid was transfected with Lipofectamine 2000 (cat. n.: 11668‐027, Invitrogen) according to the manufacturer's instructions. The cells were reseeded to the required plate or dish 24 h after transfection. Treatments were started 72 h after transfections.

### 2.4 Stable cell line construction

Plasmid with TurboGFP‐tagged MT‐2A (cat. no: RG202748, OriGene) was transfected with Lipofectamine 2000 (cat. no: 11668‐027, Invitrogen) according to the manufacturer's instruction. Cells were further cultivated in the presence of selective antibiotics (Geneticin, G418; Sigma, 400 μg·mL^−1^). Medium with Geneticin was replaced every 2–3 days until the population of resistant cells was fully established. Clonal cell lines were further produced from single cells.

### 2.5 XTT assay

5000 cells were seeded into a 96‐well plate. The cells were treated as indicated in figure annotation. 72 h (24 h for RPE‐1) after treatment, an XTT assay (AppliChem, Darmstadt, Germany) was performed according to the manufacturer’s instructions. Briefly, XTT solution was added to media and incubated for 30–120 min. The dye intensity was measured at the 475 nm wavelength using a spectrometer (TECAN, Infinite M200PRO). Results are shown as mean values and standard deviations from 3 independent experiments, each performed in 5 technical replicates.

### 2.6 Cell fractionation

Cells were treated as indicated in figure annotation. Before harvesting, the cells were washed by cold PBS. Lysis buffer (50 mm HEPES, pH 7.4, 150 mm NaCl, 2 mm MgCl2, 10% glycerol, 0.5% Triton X‐100, and protease inhibitor cocktail by Roche) was applied to the cells and kept for 10 min gently agitating at 4 °C. After that, cells were scraped and the whole mixture was placed inside Eppendorf tubes and kept for another 10 min on ice with intermittent vortexing. After that, the mixture was centrifuged at 20 000× ***g*** for 10 min at 4 °C. Insoluble fraction and supernatant were each separately diluted in 2x LSB buffer and used for western blot analyses.

### 2.7 Western blot

Equal amounts of cell lysates were separated by SDS/PAGE on hand‐cast (8%, 15%) or commercial gradient 4–15% Mini‐PROTEAN TGX Precast Gel (cat. no: 4561083 or 4561086, BIO‐RAD). Separated proteins were transferred onto a nitrocellulose membrane. The membrane was blocked with 5% bovine milk in Tris‐buffered saline containing 0.1% Tween 20 for 1 h at room temperature and then incubated overnight at 4 °C with primary antibodies followed by detection by secondary antibodies. Secondary antibodies were visualized by Immobilon Forte Western HSR Substrate (cat. no: WBLUF0500, Merck Millipore, Burlington, MA, USA), and images were acquired by ChemiDoc imaging system (Bio‐Rad, Hercules, CA, USA).

### 2.8 Immunofluorescence staining and quantitative microscopy

For microscopy, the cells were grown on glass coverslips. Cells were treated as indicated in figure annotation. Before fixation, the cells were pre‐extracted with pre‐extraction buffer (0.5% Triton X‐100) for 1 min, after that quickly washed by PBS, and then immediately fixed with 4% formaldehyde for 15 min at room temperature. Alternatively, the pre‐extraction step was bypassed and cells were directly fixed with 4% formaldehyde for 15 min and then permeabilized by 0.5% Triton X‐100 for 20 min. In both protocols, nuclei were stained by DAPI (1 μg·mL^−1^) at room temperature for 5 min. Samples were visualized and acquired using fluorescence microscopes (Zeiss LSM780 or Olympus IX81 ScanR automated microscope). Quantitative analysis of microscopic data was performed in scanr Analysis software. Acquired and ScanR processed data were further statistically tested in the STATISTICA 13 (TIBCO).

### 2.9 Quantitative polymerase chain reaction

The qPCR was performed in a 96‐well plate or 8‐tube strip (Roche, Basel, Switzerland). Reactions were performed in LightCycler Nano (Roche), LightCycler 480 Instrument II (Roche), or 7500 Fast Real‐Time PCR System (Applied Biosystems, Waltham, MA, USA) with a ‘gb SG PCR Master Mix’ (cat. no: 3005, Generi Biotech) or ‘Fast SYBR Green Master Mix’ (cat. no: 4385612, Applied Biosystems). Following primers were used: HSPA1A forward 5′‐GCCTTTCCAAGATTGCTGTT‐3′; reverse 5′‐TCAACATTGCAAACACAGGA‐3′ [20]; *MT‐1E* forward 5′‐GCCTGACTGCTTGTTCGTCT‐3′; reverse 5′‐AAGAGCAGTTGGGGTCCATT‐3′; *MT‐2A* forward 5′‐CCCGCTCCCAGATGTAAAGA‐3′; reverse 5′‐TAGCAAACGGTCACGGTCAG‐3′; *GAPDH* [21] forward 5′‐AGCCACATCGCTCAGACAC‐3′; reverse 5′‐GCCCAATACGACCAAATCC‐3′. Gene expression was evaluated by the delta–delta CT method.

### 2.10 Measurement of CuET in culture medium and cells

To measure the formation of CuET in culture medium, a complete cell culture medium (DMEM, 10% FBS, 1% penicillin/streptomycin) was incubated with CuET or CuET + CBD combination as described. After incubation, the medium was vortexed and mixed with acetone in a ratio of 1 : 4. The mixture was centrifuged 18 000× ***g*** for 2 min at 4 °C. The supernatant was transferred into glass HPLC vials for measurement. The CuET complex was analyzed by the HPLC‐MS method described previously [10]. The quantification of the CuET complex was calculated according to the calibration curve.

To measure the concentration of CuET in cells, subconfluent U‐2‐OS cell culture was treated with CuET or CuET + CBD combination as described. After incubation, the medium was removed, cells were washed twice with PBS, and PBS was thoroughly aspirated. Cells were scraped and stored at −80 °C. Cellular pellets were then homogenized with acetone and centrifuged 18 000× ***g*** for 2 min at 4 °C, and supernatant was transferred into a glass HPLC vial. The CuET complex was analyzed by the HPLC‐MS method described previously [10]. The quantification of the CuET complex was calculated according to the calibration curve.

### 2.11 Statistical analysis

Separated bar graphs of qPCR experiments are plotted as mean ± SD presenting 3 independent experiments. XTT assay with XY graphs comprising error bars is plotted as mean and error ± SD. All the figures represent 3 independent experiments with each point presenting 5 replicates. 2D box plots of the quantitative microscopy analysis are plotted as median ± SD. All the experiments were done in 3 independent experiments. The figure depicting the experiment represents a random selection from one of the experiments. Statistical significance was assessed by unpaired t‐test, and the resulting *P*‐value is shown in graphs and particular figure legends. The graphical processing was performed in Statistica 13 or graphpad Prism 8.0.1. Statistical significance, as well as IC50 values, was calculated in graphpad Prism 8.0.1 and 9.2.0

### 2.12 Chemicals and antibodies

The following antibodies were used for immunoblotting: anti‐β‐actin (1 : 1000; Santa Cruz Biotechnology, cat. no: sc‐47778), anti‐DDK (1 : 1000; OriGene, cat. no: TA50011‐100), anti‐GAPDH (clone 1D4, 1 : 500; GeneTex, cat. no: GTX78213), antihistone H3 (1 : 2000; Cell Signaling, cat. no: 4499P), anti‐MTF1 (1 : 1000; NOVUS Biologicals, cat. no: NBP1‐86380), anti‐NPLOC4 (1 : 1000; NOVUS Biological, cat. no: NBP1‐82166), anti‐Ubiquitin K48 (clone Apu 2, cat. no 05‐1307, Millipore) goat‐anti mouse IgG‐HRP (1 : 1000; GE Healthcare, NA931, Chicago, IL, USA), goat‐anti‐rabbit (1 : 1000; GE Healthcare, NA934), and donkey‐anti goat IgG‐HRP (Santa Cruz Biotechnology, sc‐2020, Dallas, TX, USA). The formulation of CuET (bis‐diethyldithiocarbamate‐copper complex) in water was based on direct synthesis in the presence of 1% bovine serum albumin as described previously [10, 22]. Briefly, 10 mL of 2.8 mm CuET is prepared by adding 200 μL of 280 mm solution of sodium bis‐diethyldithiocarbamate trihydrate (DTC, Sigma‐Aldrich) and 28 μL of 1 m CuCl2 (Sigma‐Aldrich) into 9772 mL of 1% solution of bovine serum albumin in ddH20 (Sigma‐Aldrich). The resulting CuET formulation was kept at 4 °C for no longer than a month. All solutions were sterile‐filtered before synthesis. The chelator bathocuproine disulfonic acid (Sigma‐Aldrich) was used for copper chelation in a final concentration of 10 μm and was added to the sample just before the disulfiram (Sigma‐Aldrich) treatment. CBD [‐(‐)cannabidiol] was ordered from Abcam (cat. n.: ab120448). 10 mm stock solution was prepared in methanol (Penta).

## 3 Results

### 3.1 NPL4‐GFP cell reporter‐based screen implicates CBD in resistance to CuET

**Fig. 1:**
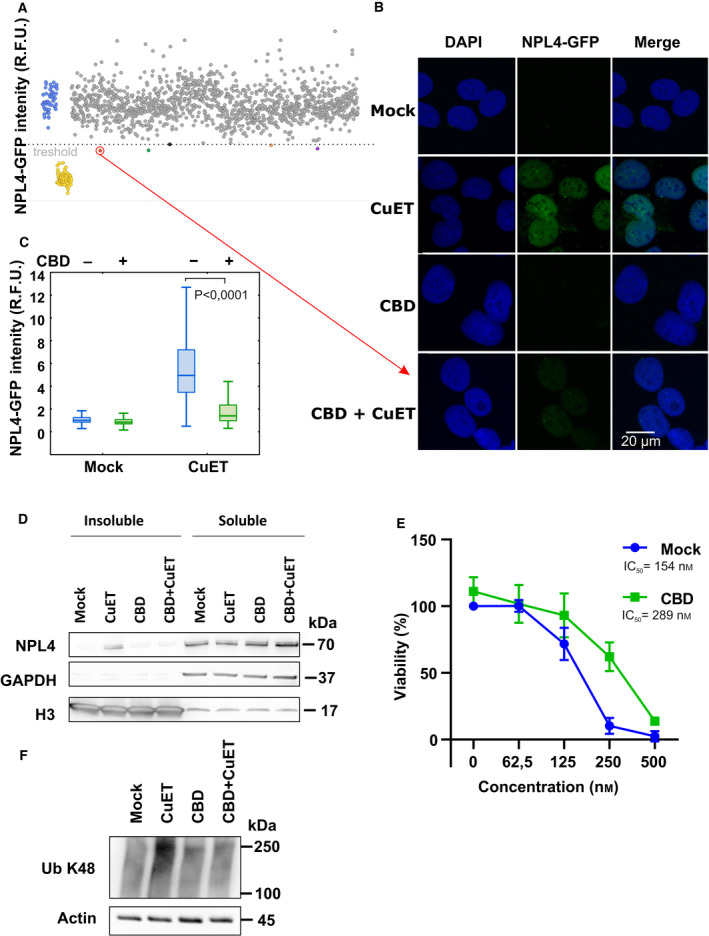
Cannabidiol (CBD) protects cells from bis‐diethyldithiocarbamate‐copper complex (CuET). (A) Dot plot depicting the results of high‐throughput screening of the chemical library. CuET + mock‐treated controls are in blue, and untreated controls are in yellow. Tested compounds overcoming the threshold of 0.5% positive hits are highlighted in red, green, black, orange, and violet colors. Cells were pretreated with compounds (10 µm) for 17 h and treated with 0.2 µm CuET for 3 h. The screening result represents one experiment (*n* = 1). (B,C) Microscopy‐based confirmation of the strongest hit. Cells were pretreated with 10 μm CBD for 17 h and treated with 0.2 μm CuET for 3 h and analyzed by microscope including microscopy‐based quantitative analysis (20 µm scale bar) of NPL4‐GFP signal in Triton X‐100 pre‐extracted cells. Combined treatment shows significantly less accumulation of nondissolvable (pre‐extraction resistant) NPL4 protein compared with CuET treatment only (two‐tailed *t*‐test). Pre‐extraction was performed before the fixation step. The figures show results from one of three independent experiments (*n* = 3). (D) Cells pretreated with 10 μm CBD for 17 h and treated with 0.2 μm CuET for 3 h accumulate less endogenous NPL4 protein in insoluble fractions compared with CuET treatment only as observed by western blot (WB). The figure shows one of three independent experiments (*n* = 3). (E) Cells pretreated with 10 μm CBD for 17 h and treated with increasing concentration of CuET for 72 h are more resistant compared with CuET treatment only as observed by the XTT assay. The results represent the mean and standard deviation of three independent experiments (*n* = 3). (F) WB analysis of K48 polyubiquitinated (Ub K48) proteins reflecting differences in a malfunction of protein degradation in mock, CuET, CBD, and CBD + CuET‐treated cells. For the experiment, the cells were treated for 3h by 0.2 μm CuET. In the combined treatment, CuET was added 17 h after 10 μm CBD. The figure shows one of three independent experiments (*n* = 3).

We set up a high‐throughput screening approach for the identification of CuET sensitivity modulators. The screen was based on a reporter human sarcoma U‐2‐OS cell line expressing GFP‐tagged NPL4 protein, the molecular target of CuET’s anticancer effects. Upon CuET treatment, NPL4 undergoes robust aggregation and immobilization, a phenotype exploited in our screen to search for NPL4‐GFP fluorescence in the insoluble cell fraction [10]. Immobilized NPL4‐GFP can be quantified using microscopy‐based analysis after detergent (Triton X‐100) pre‐extraction of drug‐exposed cultured cells. The pre‐extraction procedure washes away soluble proteins from cells, thereby selectively enriching for insoluble proteins including the aggregated NPL4. For the screening setup, we pretreated cells with various chemicals from our library of 1282 pharmacologically active compounds overnight at the nontoxic concentration (for the list of compounds used in the screen, Table S1). The next day, the cells were exposed to 0.2 μm of CuET for 3 h, as the latter treatment leads to the insoluble aggregate formation of NPL4 and consequently to pre‐extraction‐resistant NPL4‐GFP signal conveniently detected by high‐throughput microscopy. Interestingly, several compounds substantially decreased the level of such CuET‐immobilized NPL4‐GFP signal suggesting possible interference with CuET treatment. Cannabidiol (CBD), the nonpsychotropic component of marijuana, was the strongest hit in our screen (Fig. 1A), moreover a hit with high clinical relevance due to its rather common use among cancer patients [23].

The CBD hit was then further validated by more detailed microscopy‐based analysis in the U‐2‐OS‐NPL4‐GFP cell line (Fig. 1B,C). The same CBD‐promoted rescuing effect from CuET‐evoked aggregation of NPL4 was confirmed also for the endogenous NPL4 protein using immunoblotting analysis. Thus, CuET administered alone promotes accumulation of NPL4 within the nondissolvable cellular fraction, an effect that was reduced in two cell lines, U‐2‐OS osteosarcoma and breast cancer‐derived MDA‐MB‐231 when exposed to a combined CBD + CuET treatment (Fig. 1D, Fig. S1A). Next, we addressed whether the reduced aggregation of NPL4 by CBD affects also the CuET cancer cell toxicity profile. To this end, the cells were pretreated by CBD overnight and then treated with CBD along with increasing concentrations of CuET for 72 h. The significant rescue effect of CBD in terms of better cell survival was confirmed for both tested cell lines (Fig. 1E and Fig. S6C as part of the following siRNA combined experiments). Both cell lines pretreated by CBD also displayed decreased accumulation of K48 polyubiquitylated (poly‐Ub) proteins, a surrogate marker for impaired protein degradation caused by malfunction of the p97‐NPL4 segregase pathway [10] (Fig. 1F, Fig. S1B). The activation of the heat‐shock pathway is yet another marker of CuET‐induced proteotoxic stress [10] the effect of which can be quantified by qPCR by examination of mRNA levels of HSPA1A, the major stress‐inducible member of the HSP70 family. Consistently, CuET highly induced HSPA1A mRNA levels which effect was significantly decreased in cells pretreated by CBD (Fig. S2A). These results indicate that CBD attenuates the CuET‐promoted aggregation of NPL4 resulting in reduced toxicity of this compound toward cancer cells.

### 3.2 Both CBD and CuET induce expression of the metallothionein family members

To explore the mechanism of the rescue effect described above, we first tested for a potential direct interaction of CBD with CuET and/or reduced uptake of CuET as the most straightforward explanations. To address this possibility, we used the HPLC‐MS‐based detection allowing direct monitoring of the CuET levels in cells [10]. We detected similar levels of CuET in control and CBD‐pretreated cells, suggesting that the observed drug interference in the CBD pretreated cells is unlikely to be attributable to a lower cellular uptake of CuET (Fig. S3A, B).

Available literature describes various cellular responses to CBD treatment [12, 13, 14, 15] including activation of the so‐called metallothionein pathway [24, 25, 26]. This particular pathway might plausibly explain the rescuing effect from the CuET‐evoked NPL4 aggregation observed in our experiments as the proteins in the family of metallothioneins (MTs) are rich in cysteines that can chelate divalent metals via sulfhydryl groups. Metals that avidly bind to MTs include Cd, Zn, and Cu which are either used, stored, or removed from the cell [27]. The CuET molecule contains noncovalently bound divalent copper, and thus, MTs might ‘neutralize’ this compound as part of their known toxic ion detoxification function [28].

**Fig. 2:**
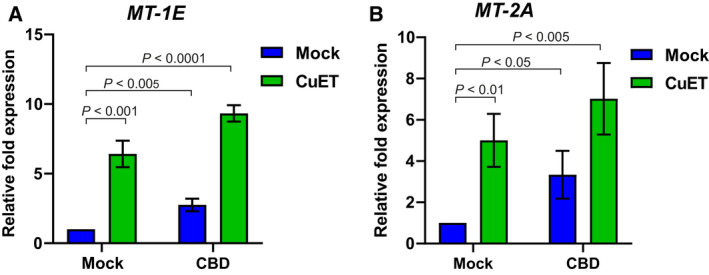
Induction of metallothioneins *MT‐1E*, *MT‐2A* expression by cannabidiol (CBD), and bis‐diethyldithiocarbamate‐copper complex (CuET) measured by quantitative polymerase chain reaction (qPCR) A) CBD and CuET increase the expression of *MT‐1E* mRNA. B) CBD and CuET increase the expression of *MT‐2A* mRNA. The experimental setup involved pretreatment with 10 μm CBD for 17 h and treatment with 0.2 µm CuET for 3 h. In the combined treatment, CuET was added 17 h after CBD. For both charts, a two‐tailed t‐test was used for *P*‐value calculation. The result represents the mean and standard deviation of three independent experiments (*n* = 3).

**Fig. 3:**
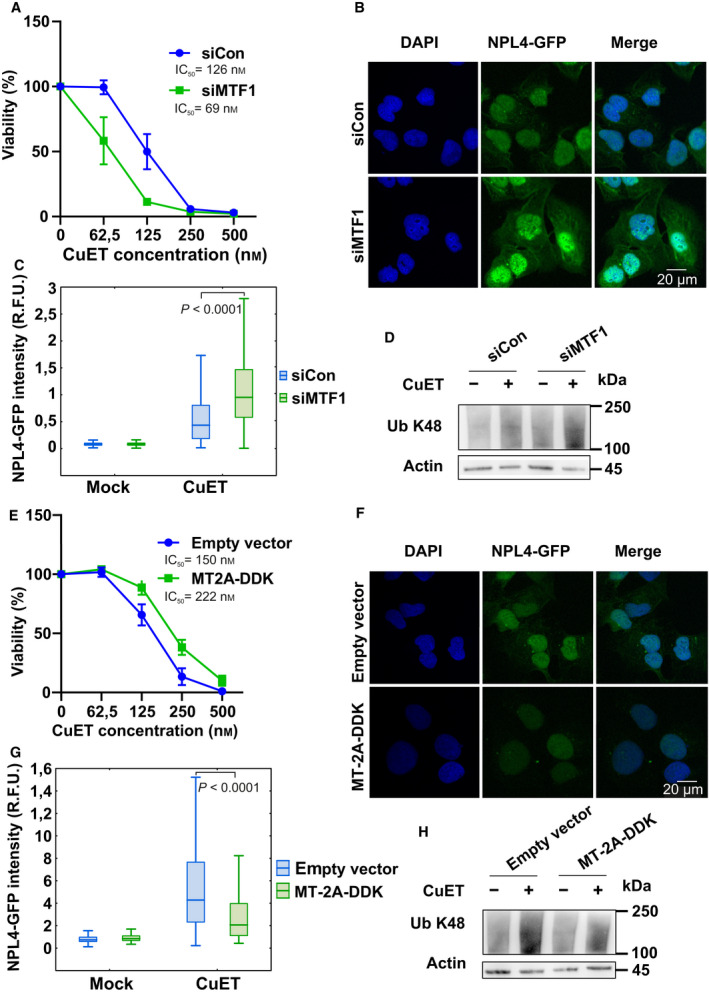
Metallothionein level modulates the cellular responses to bis‐diethyldithiocarbamate‐copper complex (CuET) (A) MTF1‐silenced cells are more sensitive to CuET. Cells were treated with increasing concentration of CuET for 72 h and analyzed by XTT assay. The result represents the mean and standard deviation of three independent experiments (*n* = 3). (B, C) MTF1‐silenced cells accumulate significantly more of the nondissolvable NPL4 after CuET as depicted by microscopic images (20 µm scale bar) and corresponding quantitative microscopic analysis of NPL4‐GFP signal in Triton X‐100 pre‐extracted cells. Cells were treated with 0.2 μm CuET for 3 h. A two‐tailed t‐test was used for *P*‐value calculation. The results represent one of three independent experiments (*n* = 3). (D) MTF1‐silenced cells accumulate more K48 polyubiquitinated (Ub K48) proteins after CuET treatment. Cells were treated with 0.2 μm CuET for 3 h and analyzed by western blot (WB). The figure shows one of three independent experiments (*n* = 3). (E) MT‐2A‐overexpressing cells are more resistant to CuET. Cells were treated with increasing concentrations of CuET for 72 h and analyzed by XTT assay. The result represents the mean and standard deviation of three independent experiments (*n* = 3). (F, G) MT‐2A‐overexpressing cells accumulate significantly less of the nondissolvable NPL4 as depicted by microscopic images (20 µm scale bar) and corresponding quantitative microscopic analysis of NPL4‐GFP signal in Triton X‐100 pre‐extracted cells. Cells were treated with 0.2 μm CuET for 3 h. A two‐tailed t‐test was used for *P*‐value calculation. The results represent one of three independent experiments (*n* = 3). (H) MT‐2A‐overexpressing cells accumulate fewer K48 polyubiquitinated proteins after CuET. Cells were treated with 0.2 μm CuET for 3 h and analyzed by WB. The figure shows one of three independent experiments (*n* = 3).

**Fig. 4:**
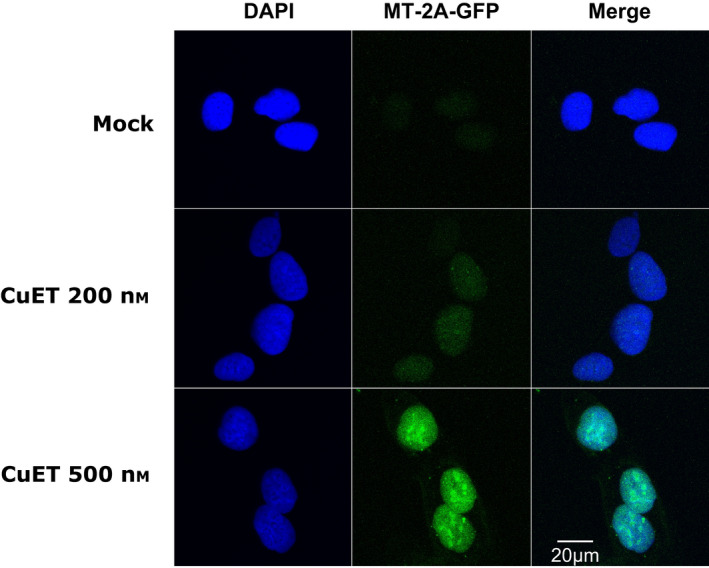
Bis‐diethyldithiocarbamate‐copper complex CuET treatment immobilizes MT‐2A‐GFP. MT‐2A‐GFP protein is immobilized after CuET treatment revealed as depicted by microscopic images after Triton X‐100 based pre‐extraction. Cells were treated with 0.2 and 0.5 μm CuET for 3 h (20 µm scale bar). The figure represents one of two independent experiments (*n* = 2).

To test the hypothesis that MT overexpression may explain the observed rescue effect, we first confirmed that CBD indeed induces the expression of metallothioneins using quantitative polymerase chain reaction (qPCR). The mRNA levels of *MT‐1E* and *MT‐2A* roughly doubled after overnight CBD treatment in U‐2‐OS cells (Fig. 2A,B, Fig. 4A,B). Interestingly, treatment by CuET for 3 h also evoked a robust increase in the mRNA levels for *MT‐1E* and *MT‐2A*, indicating that CuET can rapidly trigger this cellular heavy metal defense mechanism. In the combined treatment with CBD and CuET, using drug concentrations that parallel those used in our phenotype rescue experiments, the induced expression levels of the MTs were even higher than after exposure to either compound alone (Fig. 2A,B, Fig. 4A,B).

### 3.3 CBD‐induced metallothioneins protect cells from CuET‐mediated toxicity

**Fig. 5:**
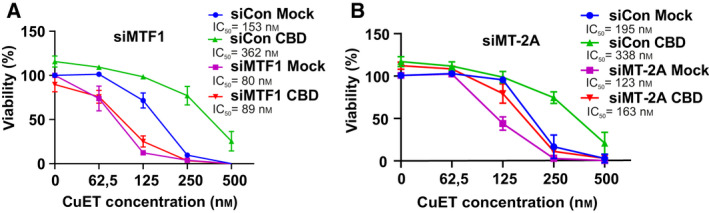
Concomitant gene silencing with cannabidiol (CBD) treatment and role of metallothionein MT‐2A in response to bis‐diethyldithiocarbamate‐copper complex (CuET) analyzed by XTT assay (A) CBD pretreatment does not protect MTF1‐silenced cells from CuET toxicity. Cells were pretreated with 10 μm CBD for 17 h and treated with 10 μm CBD and increasing concentration of CuET for 72 h. The result represents the mean and standard deviation of three independent experiments (*n* = 3). (B) MT‐2A‐silenced cells are sensitized to CuET treatment. CBD pretreatment protects MT‐2A‐silenced cells from CuET toxicity only partially. Cells were pretreated with 10 μm CBD for 17 h and treated with 10 μm CBD and increasing concentration of CuET for 72 h. The result represents the mean and standard deviation of three independent experiments (*n* = 3).

Given the observed induction of metallothioneins, we next aimed at obtaining more mechanistic insights into the interplay among CBD, CuET, and MT’s. Our strategy was to directly manipulate the metallothionein pathway and assess any impact in terms of potential modulation of the CuET‐mediated cellular response. It is known that metal transcriptional factor 1 (MTF1) plays a pivotal role in MT gene expression [29]. Indeed, after the knockdown of MTF1 (Fig. S5A), we observed a significantly reduced ability of our model cell lines to induce expression of *MT‐1E* and *MT‐2A* after CuET exposure (Fig. 5B,C). Importantly, such experimentally achieved MTF1 insufficiency rendered the U‐2‐OS and MDA‐MB‐231 cells particularly sensitive to CuET treatment as confirmed in a 72‐h XTT assay (Fig. 3A, Fig. S6A,B). This hypersensitivity was accompanied by an elevated amount of immobilized NPL4‐GFP in the U‐2‐OS NPL4‐GFP reporter model (Fig. 3B,C). This is an important finding which directly links the increased toxicity with CuET's primary cellular target. The toxic effect was further underlined also by the increased accumulation of poly‐Ub proteins and increased expression of HSPA1A (Fig. 3D, Fig. S6D, Fig. S2B).

Next, we used a complementary approach and designed a cell line transiently overexpressing the DDK‐tagged (Flag‐tagged) MT‐2A protein (Fig. S5A). This cellular model was more resistant to the CuET treatment compared with the control, empty vector‐transfected cells (Fig. 3E). Cells expressing the ectopic MT‐2A also showed less immobilized NPL4‐GFP (Fig. 3F,G) under otherwise standard CuET treatment conditions, and the rescue effect was further underlined by the decreased accumulation of poly‐Ub proteins and decreased expression of HSPA1A (Fig. 3H, Fig. S2C). Next, we established a stable U‐2‐OS‐MT‐2A‐GFP cellular model to see any potential direct effects of CuET on MT‐2A protein behavior. Indeed, we could detect MT‐2A‐GFP signal immobilization (i.e., resistance to pre‐extraction) resembling the effect of CuET on NPL4‐GFP protein, suggesting the direct interaction of MT‐2A with CuET (Fig. 4).

To link the observed phenotypes more thoroughly with the CBD’s mode of action, we also tested the rescue effect in MTF1‐silenced cells. As expected, in the MTF1‐silenced cells, the rescue effect of CBD treatment on the CuET‐evoked phenotypes became negligible in both the U‐2‐OS and MDA‐MB‐231 cell lines (Fig. 5A, Fig. S6C). Analogous results were obtained also for primary human RPE‐1 cells (Fig. S7A, B). Besides MTF1 knockdown, also direct silencing of *MT‐2A* (Fig. S5D) was similarly able to render the U‐2‐OS cells more sensitive to CuET treatment (Fig. 5B).

Our findings that high levels of metallothioneins in tumor cells can cause resistance to CuET, while cancer cells harboring low‐level MT’s may be more sensitive to such treatment, raise a possibility that metallothionein expression levels might help predict responses to CuET (DSF) treatment in the future. One prediction for such candidate biomarker application is that the starting endogenous expression levels of MTs would vary among individual models or clinical specimens, preferably showing at least a subset of cases with expression levels below those in corresponding normal cell/tissue type. As the first step toward testing the landscape of MT expression patterns, we employed qPCR to assess mRNA levels of two relevant MTs: MT‐1E and MT‐2A, among a panel of 11 human cell lines derived from diverse types of breast cancer (2 luminal, 2 HER‐positive, and 7 triple‐negative, see Methods), compared with levels found in the nontransformed human MCF 10A cells as a reference (Fig. S8). Notably, the expression of both MT‐1E and MT‐2A was more than an order of magnitude lower in the majority of these cancer cell models compared with MCF 10A cells, except for triple‐negative cell lines, some of which expressed levels comparable with those in the control MCF 10A (Fig. S8). None of the 11 cancer cell lines showed levels of either MT that would exceed expression seen in the MCF 10A control.

Altogether, these results show that CBD induces the metallothionein pathway consistently in various cellular backgrounds, that this cellular response leads to enhanced MT expression which protects cancer cells against the DSF’s anticancer metabolite CuET, and that low levels of MTs render cancer cells more sensitive to CuET treatment, raising a possibility to explore MT levels as candidate biomarkers for future clinical applications.

## 4 Discussion

In this work, we show how the high‐throughput screening approach combined with the high content microscopy analysis can be used for addressing highly relevant clinical issues. By setting up the phenotypic screening involving the known drug target (NPL4) as the readout, we were able to identify a clinically relevant compound—cannabidiol (CBD), as the most likely cause of unwanted interference with ongoing cancer treatment with disulfiram (Antabuse), the anticancer effects of which are currently tested in multiple ongoing preclinical studies and clinical trials [30]. Furthermore, our present experiments also reveal the mechanistic basis of this CBD‐mediated interference. It is known that DSF targets cancer cells via its direct metabolite CuET [10], chemically bis‐diethyl‐dithiocarbamate‐copper complex. Inside cells, CuET binds and aggregates NPL4, an important factor for protein processing and degradation [10]. Concomitant treatment with CBD induces overexpression of metallothioneins which compete with NPL4 for the available CuET, thereby ultimately lowering the efficacy of treatment by CuET.

CBD, the nonpsychotropic component of marijuana has become the focus of attention in medicine in recent years. Numerous studies have revealed the considerable potential of the substance for patients with diseases of the nervous system, inflammatory diseases, or cancer. In several countries, marijuana is now accepted as a medical drug, and CBD itself is sold with or without prescription in various forms. An example of the prescription‐available form is Epidiolex used for the treatment of seizures in two types of epilepsy and tuberous sclerosis complex in the United States. In the context of this study, it is important that CBD and various cannabis products are becoming popular among cancer patients due to their potential to mitigate chemotherapy‐induced side effects including chronic pain, nausea, vomiting, loss of appetite, and anxiety [16, 17].

Interestingly, the potential of MTs as detoxifying proteins has been known for decades and this function has also been linked to possible resistance to some chemotherapeutics [31, 32, 33]. The detoxifying ability of MTs has been reported even for some nonmetal‐based drugs. Chemotherapeutics that are sensed and bound by MTs are neutralized before reaching their intended target(s) and thereby become clinically ineffective. Thus, MT expression represents potential predictive biomarkers of resistance to specific treatments [34, 35]. In light of these facts and our data presented here, CBD usage might be a relevant factor to be kept in mind for cancer patients not only undergoing the trials with DSF‐repurposing therapy but also treated with some standard‐of‐care chemotherapy drugs.

Importantly, the effect of metallothioneins MT‐1E and MT‐2A and MTF1 transcription factor on the activity of DSF has been recently identified also in another independent study aimed at the high‐throughput screening of antitumor effects of known drugs using a molecular barcoding method called PRISM (profiling relative inhibition simultaneously in mixtures) [36]. Disulfiram (DSF) was one of the tested substances and the screen revealed that cells with reduced or lost expression of *MT‐1E* and *MT‐2A* genes became more sensitive to DSF. The authors of this study did not realize that it was not DSF but rather its metabolite CuET against which the MTs protect the cells. It seems the fact that CuET is spontaneously formed from DSF and copper ions in culture media is underappreciated by the scientific community [11]. Indeed, after blocking this conversion of DSF into CuET through chelation of copper ions from the cell culture media, DSF becomes a harmless molecule regardless of the MT expression status, as we also documented here (Fig. S9). Thus besides CBD, the copper availability for CuET formation during the trials with DSF will likely also represent one of the factors affecting the anticancer efficacy. For example, it is known that increased uptake of zinc negatively affects copper uptake and thus should be limited by the patients during such treatment [37]. Similarly, patients with celiac disease may be deficient in copper [38].

Yet another important aspect of our present study is the validation of the crucial role of the MT pathway in protecting cancer cells against the impact of DSF’s metabolite CuET on NPL4 protein. Despite DSF is intensively tested in several ongoing clinical trials aiming at repurposing DSF for cancer treatment, there is currently no biomarker suitable for the selection of patients who could most benefit from DSF, a fact that unfortunately highly limits the potential success of DSF treatment in oncology. Together with the Corsello *et al*. (2020) study, our present report highlights MTs and MTF1 as such candidate predictive biomarkers, which are upregulated and vary considerably among the patients as well as different cancer types [32, 39, 40, 41]. In this context, while preliminary, our findings of substantially lower MT‐1E and MT‐2A expression levels among a panel of human breast cancer cell line models compared with nontransformed breast epithelial cell control suggest that such potential biomarker application may be worth pursuing further. While speculative at present, if future tissue validation analyses confirm that endogenous metallothionein levels are much lower in cancer cells *in vivo* compared with corresponding normal tissue, such a striking difference may highlight yet another contributing factor helping to explain why disulfiram (and CuET) is toxic for tumor cells yet largely without major negative side effects and well tolerated by both experimental animals and people [1, 2, 5, 10]. Moreover, the link of MT pathway to CuET effectiveness can inspire new therapeutic approaches, which can be explored in future studies, such as a combination of DSF (and copper supplementation) with a compound APTO‐253, an experimental drug inhibiting MTF1 function currently tested in phase 1 clinical trial (NCT02267863).

## 5 Conclusions

For the first time, we linked CBD‐mediated activation of the metallothionein pathway with protection/resistance against CuET (an anticancer metabolite of DSF) which we believe is highly relevant for the ongoing clinical trials with DSF. Patients undergoing such treatment should avoid concomitant usage of CBD‐containing drugs. This finding may also provide a plausible explanation, at least in part, for some of the differential outcomes among cancer patients treated by DSF.

From a broader perspective, this discovery somewhat resembles the scenario of antioxidant supplements the increased uptake of which is also common among cancer patients and may potentially interfere with standard‐of‐care chemo‐radiotherapy [42]. Similarly, CBD may reduce the effectiveness of all treatments, for which the reactivity with metallothioneins has been studied [31, 32, 33]. Thus, this work could motivate further research on CBD and its interaction with anticancer drugs as an issue highly relevant for biomedicine in general and oncology in particular.

## Conflict of interest

MM, JB, ZS, and MH are co‐inventors on patent EP 17193240.3 and patent application EP 18199181.1, both utilizing CuET formulation into nanoparticles as an anticancer agent. Other authors declare no competing interests.

## Author contributions

JB, MM, TB, and ZS designed the experiments, interpreted the data, and write the manuscript; TB performed microscopic, qPCR, and most of the cellular experiments; MM contributed to the microscopic experiments; ZS performed mass spectroscopy experiments and the chelation cytotoxic tests; KCH contributed to the cytotoxicity tests and western blotting. JR, PJ, and MH designed, performed, and interpreted the HTS screen; DL contributed to the cytotoxicity tests. SA performed metallothionein expression analysis in breast cancer cell lines.

### Peer Review

The peer review history for this article is available at https://publons.com/publon/10.1002/1878‐0261.13114.

## Supporting information


**Fig. S1**. Cannabidiol (CBD) pre‐treatment modulates the cellular responses to bis‐diethyldithiocarbamate‐copper complex (CuET) in the MDA‐MB‐231 cell line as observed by western blot.
**Fig. S2**. Expression of heat‐shock protein *HSPA1A* after bis‐diethyldithiocarbamate‐copper complex (CuET) is modulated by cannabidiol (CBD) and metallothioneins in U‐2‐OS cell line.
**Fig. S3**. Cannabidiol (CBD) does not directly interact with bis‐diethyldithiocarbamate‐copper complex (CuET) neither affects its cellular uptake.
**Fig. S4**. Cannabidiol (CBD) and bis‐diethyldithiocarbamate‐copper complex (CuET) induce expression of *MT‐1E* and *MT‐2A* mRNA in the MDA‐MB‐231 cell line.
**Fig. S5**. MTF1 silencing affects the cellular ability to express metallothioneins.
**Fig. S6**. Metallothionein level modulates the toxic responses to bis‐diethyldithiocarbamate‐copper complex (CuET) in the MDA‐MB‐231 cell line.
**Fig. S7**. Primary cell line RPE‐1 exhibits similar drug responsiveness compared to tested cancer cell lines.
**Fig. S8**. mRNA levels of metallothioneins *MT‐1E* and *MT‐2A* in human breast cancer cell lines measured by quantitative polymerase chain reaction (qPCR).
**Fig. S9**. Metallothioneins protect cells against bis‐diethyldithiocarbamate‐copper complex (CuET) rather than disulfiram (DSF) as evaluated by XTT assay.

**Table S1**. A table summarizing data for screened compounds.

## Data Availability

Supporting data of this study are in Table S1 and Figures S1‐S9. Raw data are available from the corresponding author upon reasonable request.
