# Supplementary material for: Cannabidiol‐induced activation of the metallothionein pathway impedes anticancer effects of disulfiram and its metabolite CuET
Source: Mol Oncol. 2021 Oct 26;16(7):1541–54. doi: 10.1002/1878-0261.13114 (PMC8978514; doi:10.1002/1878-0261.13114)
Supplement: Supplementary file 1 — Fig. S1. Cannabidiol (CBD) pre‐treatment modulates the cellular responses to bis‐diethyldithiocarbamate‐copper complex (CuET) in the MDA‐MB‐231 cell line as observed by western blot. Fig. S2. Expression of heat‐shock protein HSPA1A after bis‐diethyldithiocarbamate‐copper complex (CuET) is modulated by cannabidiol (CBD) and metallothioneins in U‐2‐OS cell line. Fig. S3. Cannabidiol (CBD) does not directly interact with bis‐diethyldithiocarbamate‐copper complex (CuET) neither affects its cellular uptake. Fig. S4. Cannabidiol (CBD) and bis‐diethyldithiocarbamate‐copper complex (CuET) induce expression of MT‐1E and MT‐2A mRNA in the MDA‐MB‐231 cell line. Fig. S5. MTF1 silencing affects the cellular ability to express metallothioneins. Fig. S6. Metallothionein level modulates the toxic responses to bis‐diethyldithiocarbamate‐copper complex (CuET) in the MDA‐MB‐231 cell line. Fig. S7. Primary cell line RPE‐1 exhibits similar drug responsiveness compared to tested cancer cell lines. Fig. S8. mRNA levels of metallothioneins MT‐1E and MT‐2A in human breast cancer cell lines measured by quantitative polymerase chain reaction (qPCR). Fig. S9. Metallothioneins protect cells against bis‐diethyldithiocarbamate‐copper complex (CuET) rather than disulfiram (DSF) as evaluated by XTT assay. [file MOL2-16-1541-s002.docx]

**Supplementary Figures**

**
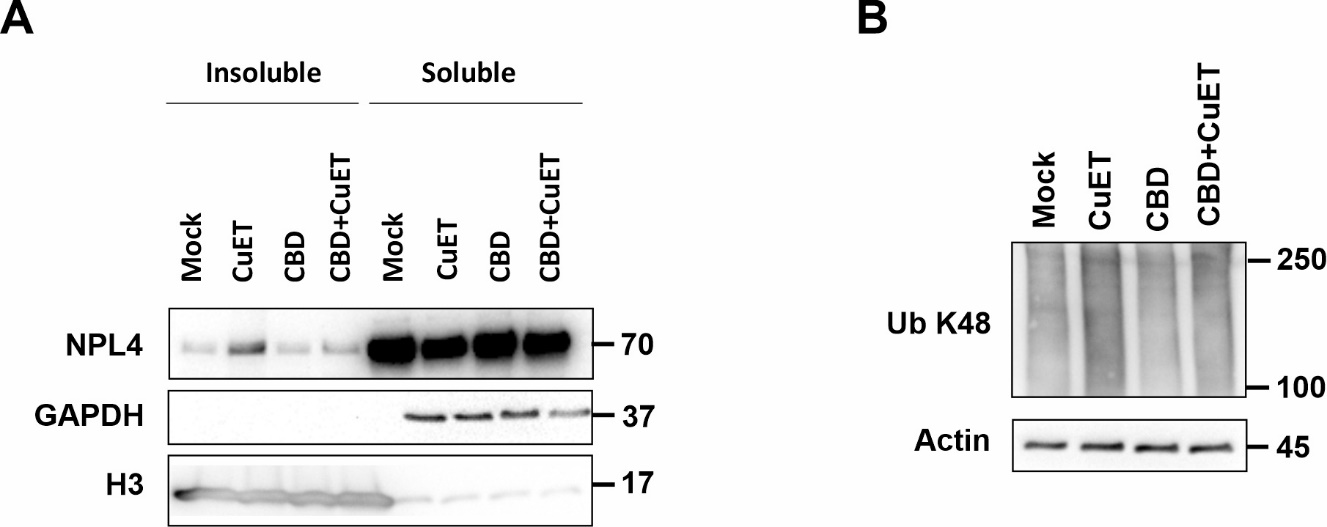
**

**Supplementary Figure 1.: Cannabidiol (CBD) pre-treatment modulates the cellular responses to bis-diethyldithiocarbamate-copper complex (CuET) in the MDA-MB-231 cell line as observed by western blot.** A) CBD pretreated cells accumulate less endogenous NPL4 protein in insoluble fractions compared to CuET treated cells alone. Cells were pretreated with 10μM CBD for 17 hours and treated with 0,2μM CuET for 3 hours. B) CBD pretreated cells accumulate fewer K-48 poly-ubiquitinated (Ub K48) proteins than CuET treated cells alone. Cells were pretreated with 10μM CBD for 17 hours and treated with 0,2μM CuET for 3 hours. The results represent one of three independent experiments (n = 3).


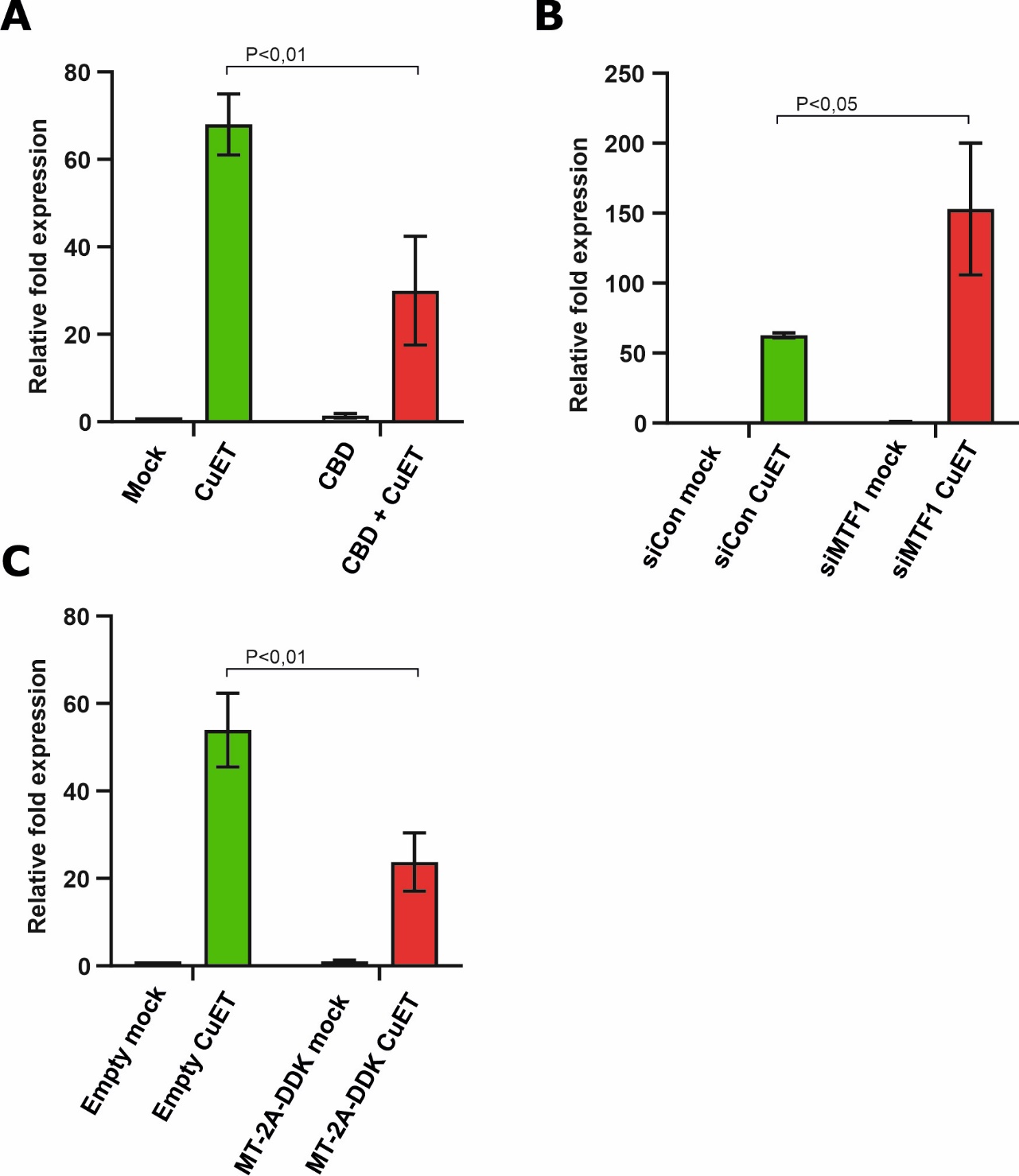


**Supplementary Figure. 2.:** **Expression of heat-shock protein *HSPA1A* after bis-diethyldithiocarbamate-copper complex (CuET) is modulated by cannabidiol (CBD) and metallothioneins in U-2-OS cell line.** A) CBD decreases CuET-induced expression of *HSPA1A.* B) Knockdown of MTF1 transcription factor increases *HSPA1A* mRNA level in CuET-treated cells. C) MT-2A-DDK overexpression decreases CuET-induced expression of *HSPA1A*. Cells were treated with 0,2μM CuET for 3 hours and evaluated by quantitative polymerase chain reaction (qPCR). T-test was used for P-value calculations. The result represents the mean and standard deviation of three independent experiments (n = 3).


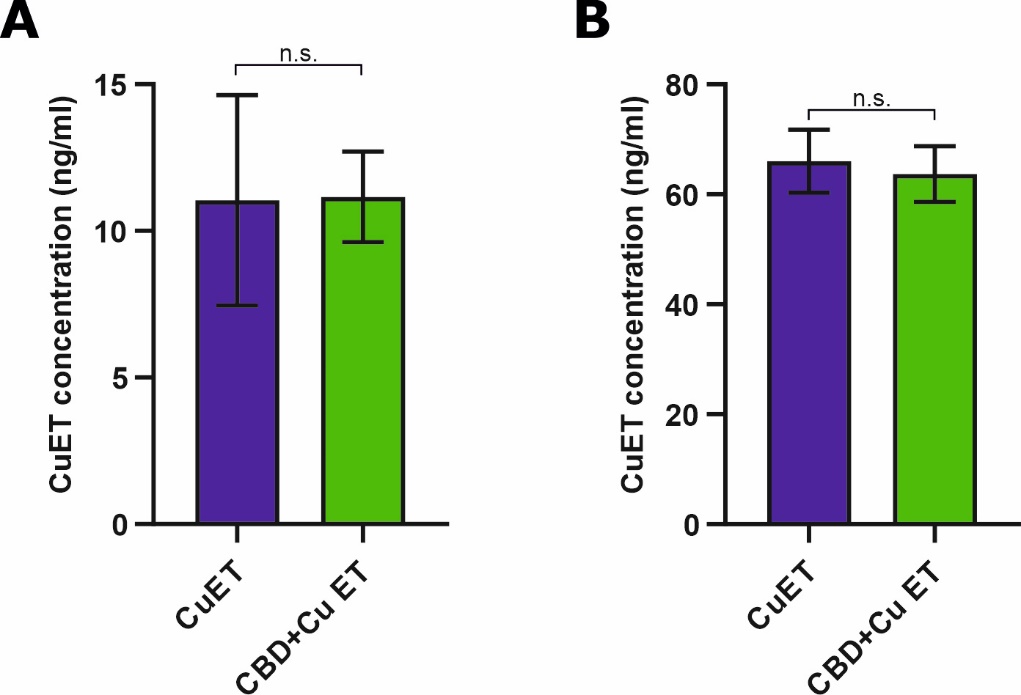


**Supplementary Figure. 3.: Cannabidiol (CBD) does not directly interact with bis-diethyldithiocarbamate-copper complex (CuET) neither affects its cellular uptake.** A) CuET levels in cells measured by high-pressure liquid chromatography combined with mass spectrometry (HPLC-MS) were maintained regardless of CBD pretreatment. Cells were pretreated with 10μM CBD for 17 hours and treated with 0,2μM CuET for 3 hours. B) CuET level in media measured by HPLC-MS is not affected by the CBD presence. 10μM CBD and 0,2μM CuET were added to the media and incubated in parallel with cells. In both cases, the difference is not significant (T-test). The results represent the mean and standard deviation of three independent experiments (n = 3).


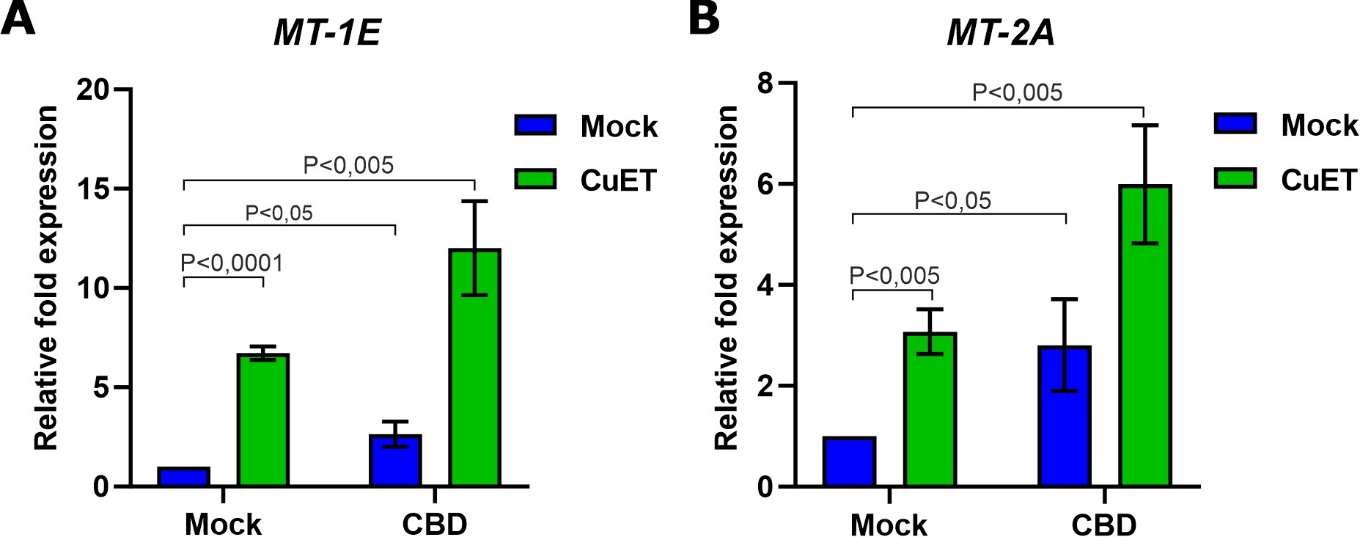


**Supplementary Figure. 4.: Cannabidiol (CBD) and bis-diethyldithiocarbamate-copper complex (CuET) induce expression of *MT-1E* and *MT-2A* mRNA in the MDA-MB-231 cell line.** A) CBD and CuET increase the expression of *MT-1E. B)* CBD and CuET increase the expression of *MT-2A*. The experimental setup involved pretreatment with 10μM CBD for 17 hours and treatment with 0,2µM CuET for 3 hours. In the combined treatment, CuET was added 17 hours after CBD. Samples were analyzed by quantitative polymerase chain reaction (qPCR). For both data sets, a T-test was used for P-value calculation. The results represent the mean and standard deviation of three independent experiments (n = 3).

**
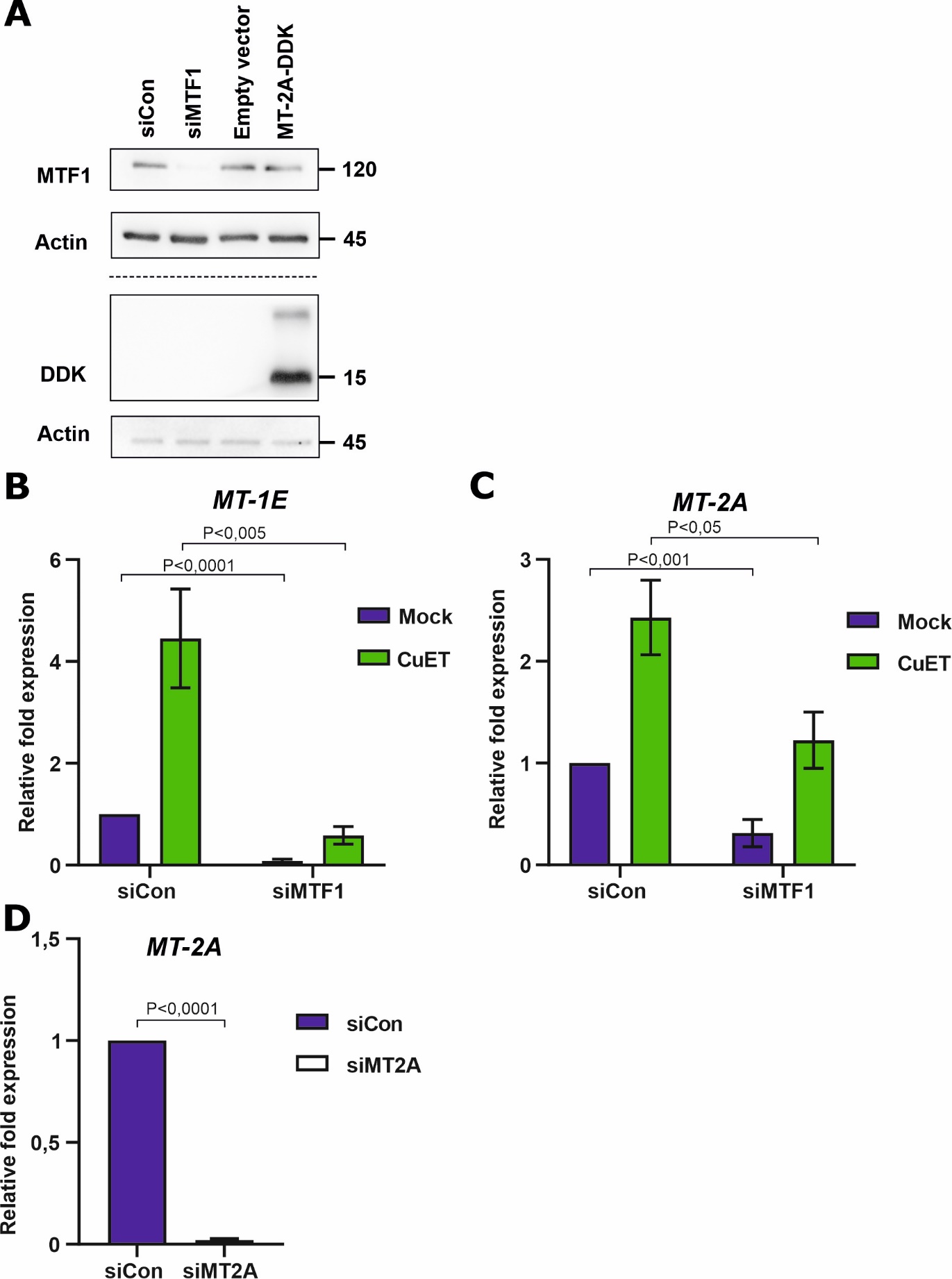
**

**Supplementary Figure. 5.: MTF1 silencing affects the cellular ability to express metallothioneins** A) Western blot (WB)-based confirmation of MTF1 and MT-2A-DDK levels in U-2-OS cells after transfection of siMTF1 and plasmid Myc-DDK-tagged MT-2A. B, C) Knockdown of MTF1 transcription factor decreases *MT-1E* and *MT-2A* mRNA levels in the mock-treated and bis-diethyldithiocarbamate-copper complex (CuET)-treated cells. Transfected cells were treated with 0,2μM CuET for 3 hours and evaluated by quantitative polymerase chain reaction (qPCR). T-test was used for P-value calculations. The result represents the mean and standard deviation of three independent experiments (n = 3). D) siRNA-based silencing of metallothionein MT-2A verified by qPCR in U-2-OS cells. T-test was used for P-value calculation. The result represents the mean and standard deviation of three independent experiments (n = 3).


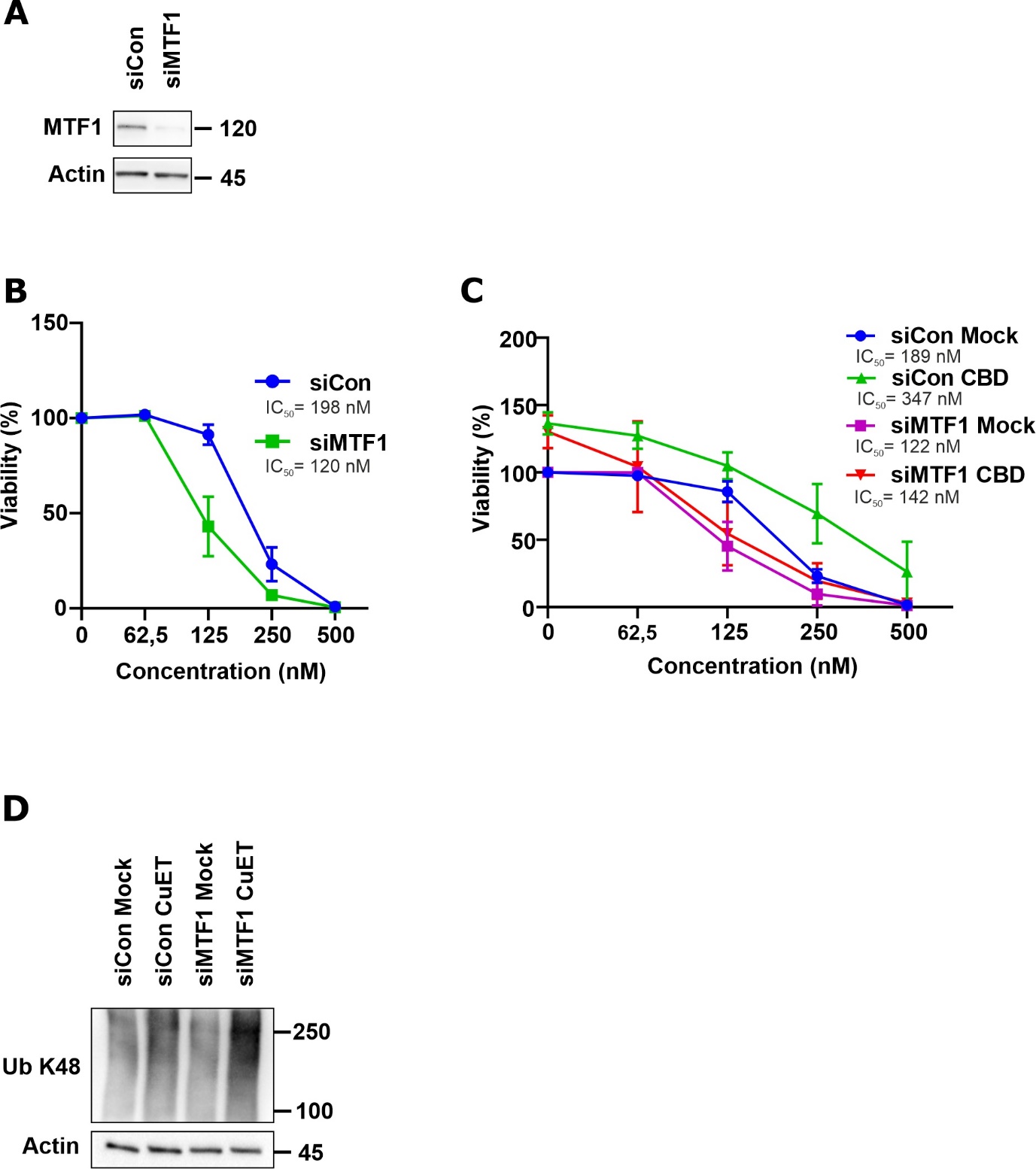


**Supplementary Figure. 6.:** **Metallothionein level modulates the toxic responses to** **bis-diethyldithiocarbamate-copper complex (CuET) in the MDA-MB-231 cell line.** A) Western blot (WB)-based verification of knockdown efficacy of the MTF1 transcription factor in the MDA-MB-231 cell line. The result represents one of three experiments B) MTF1-silenced cells are sensitized to CuET treatment. C) In MTF1*-*silenced cells cannabidiol (CBD) pretreatment does not protect cells from CuET toxicity. For both B and C experiments, the cells were pretreated with 10μM CBD for 17 hours and treated with 10μM CBD and increasing the concentration of CuET for 72 hours and evaluated by XTT assay. The result represents the mean and standard deviation of three independent experiments (n = 3). D) MTF1-silenced cells accumulate more K-48 poly-ubiquitinated (Ub K48) proteins after CuET treatment. Cells were treated with 0,2μM CuET for 3 hours and analyzed by WB. The result represents one of three independent experiments (n = 3).


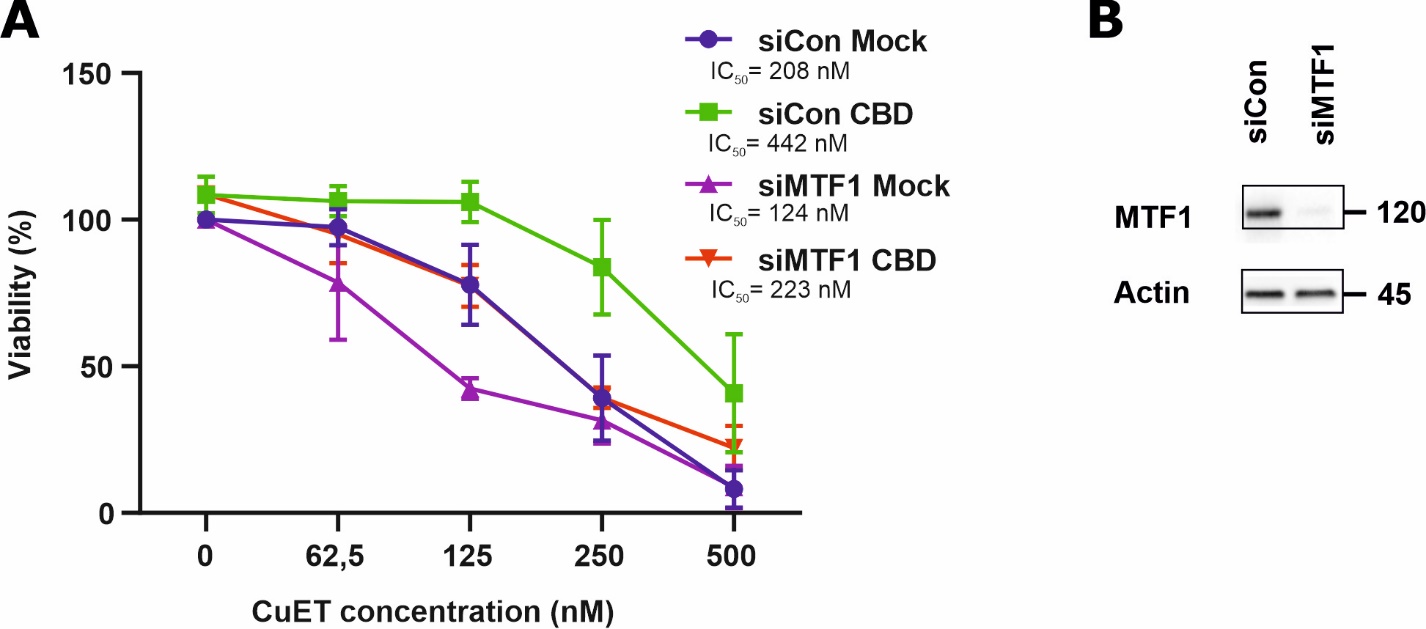


**Supplementary Figure. 7.:** **Primary cell line RPE-1 exhibits similar drug responsiveness compared to tested cancer cell lines.** A) Cannabidiol (CBD) pretreated RPE-1 cells are more resistant to bis-diethyldithiocarbamate-copper complex (CuET) treatment. MTF1-silenced RPE-1 cells are more sensitive to CuET treatment. Protection of RPE-1 cells by CBD is decreased after MTF1-silencing. Cells were pretreated with 10μM CBD for 17 hours and treated with 10μM CBD and increasing concentration of CuET for 24 hours and evaluated by XTT assay. The result represents the mean and standard deviation of three independent experiments (n = 3). B) MTF1 knockdown in RPE-1 cells verified by western blot (WB).


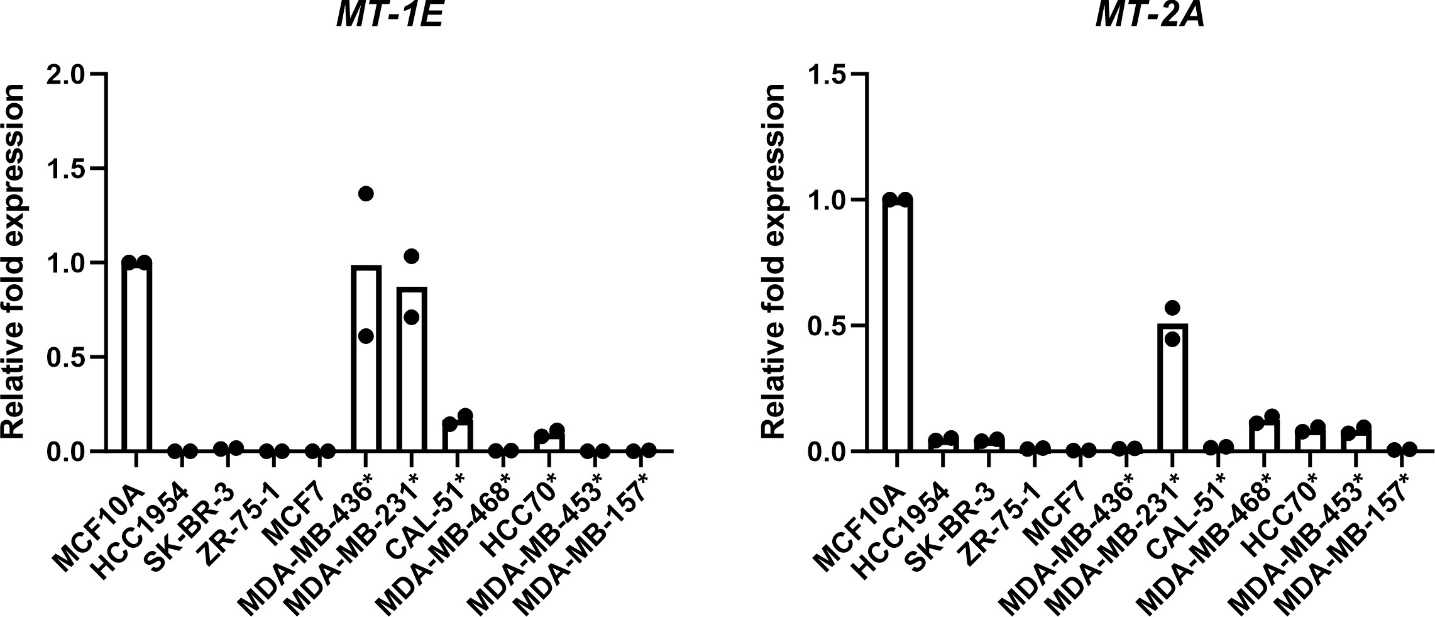


**Supplementary Figure. 8.:** **mRNA levels of metallothioneins *MT-1E* and *MT-2A* in human breast cancer cell lines measured by quantitative polymerase chain reaction (qPCR)** A) Expression of *MT-1E* in breast cancer cell lines normalized to non-transformed breast epithelial line MCF 10A. B) Expression of *MT-2A* in breast cancer cell lines normalized to non-transformed breast epithelial line MCF 10A. Cells were maintained 24 hours in fresh cell culture media before sample collection. The result represents the mean and values of two independent experiments (n = 2). Cell lines marked by an asterisk are those derived from the triple-negative breast cancer subtype.


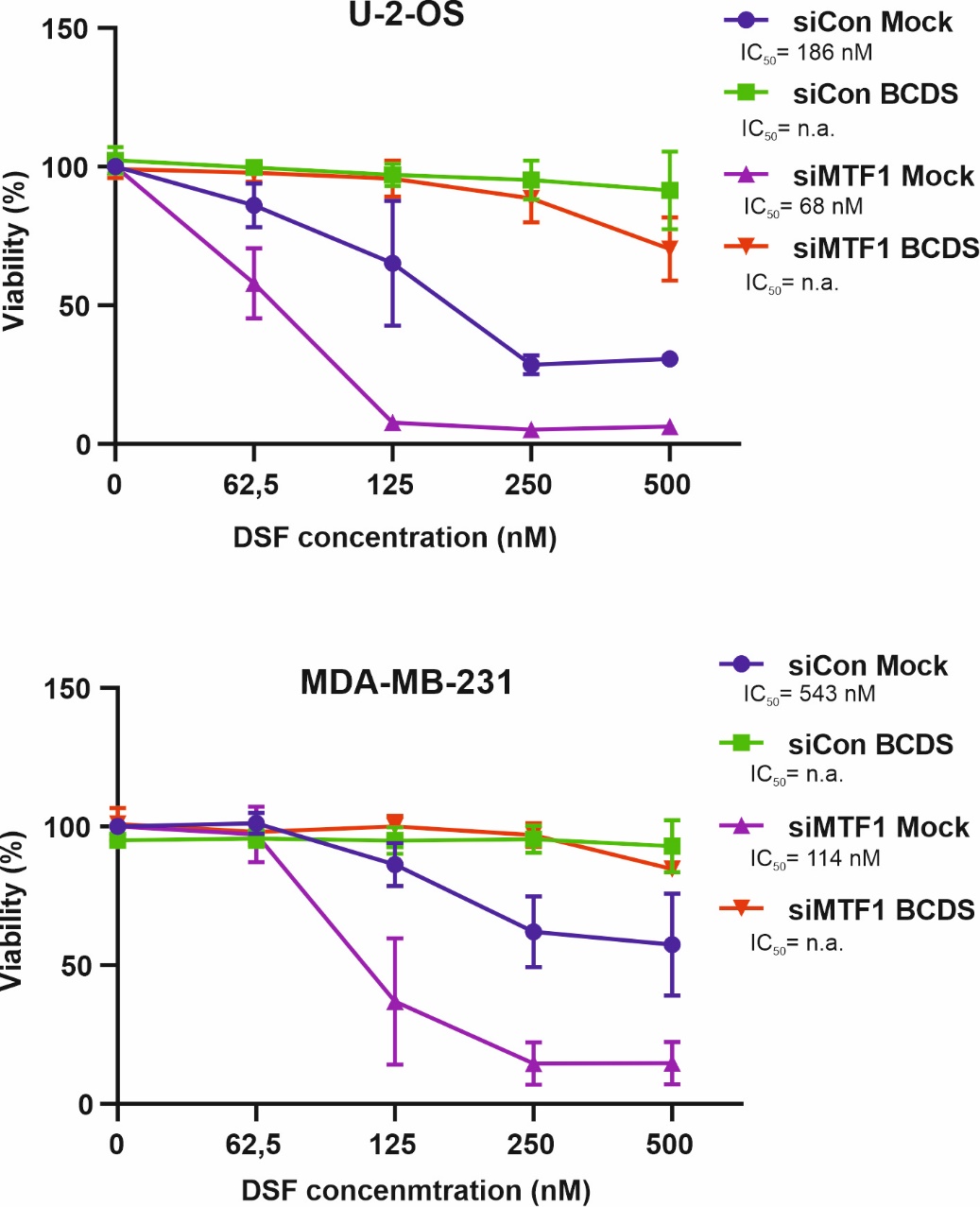


**Supplementary Figure. 9.:** **Metallothioneins protect cells against** **bis-diethyldithiocarbamate-copper complex (CuET) rather than disulfiram (DSF) as evaluated by XTT assay.** Copper ion chelation from the media containing DSF disables the formation of CuET. Thus, DSF becomes harmless/inefficient regardless of MTF1-silencing. Cell lines U-2-OS and MDA-MB-231 were treated with increased concentration of DSF and incubated for 72 hours. Copper chelator bathocuproine disulfonic acid (BCDS) was added in a final concentration of 10μM just before the DSF treatment. The result represents the mean and standard deviation of three independent experiments (n = 3).
